# A Preliminary Approach to Define the Microbiological Profile of Naturally Fermented *Peranzana Alta Daunia* Table Olives

**DOI:** 10.3390/foods11142100

**Published:** 2022-07-14

**Authors:** Barbara Speranza, Milena Sinigaglia, Maria Rosaria Corbo, Nazzario D’Errico, Antonio Bevilacqua

**Affiliations:** 1 Department of Agriculture, Food, Natural Resources and Engineering (DAFNE), University of Foggia, Via Napoli 25, 71122 Foggia, Italy; barbara.speranza@unifg.it (B.S.); milena.sinigaglia@unifg.it (M.S.); mariarosaria.corbo@unifg.it (M.R.C.); 2 Consorzio Peranzana Alta Daunia, Via Goito 8, 71017 Torremaggiore, Italy; nazder@libero.it

**Keywords:** lactobacilli, *Saccharomyces*, non-*Saccharomyces*, ratio lactic acid bacteria vs. yeasts

## Abstract

Samples of brines from *Peranzana Alta Daunia* olives at the end of fermentation were analyzed; samples were taken in two different years from eight different locations (Torremaggiore, San Severo, San Paolo di Civitate, Lucera, Chieuti, Serracapriola, Gargano and Termoli in Southern Italy). Total aerobic count, enterobacteria, pseudomonads, staphylococci, lactic acid bacteria and yeasts (*Saccharomyces* and non-*Saccharomyces*) were assessed; moreover, presumptive lactobacilli were characterized in relation to their ability to grow with salt added, and at 10 and 45 °C. Yeasts were generally more abundant than lactic acid bacteria (LAB), but two clusters were found: one including the areas of Torremaggiore, San Severo, Apricena, Lucera and San Paolo di Civitate (area 1, A1), and another comprising Gargano, Termoli and Serracapriola (area 2, A2). Lactobacilli of A1 were more resistant to stress conditions (growth at 10% of salt and at 10 °C); moreover, A1 was characterized by a lower abundance of yeasts. In some areas (Lucera and San Severo), a higher abundance of non-*Saccharomyces* yeasts was found. This paper offers a first insight into the profile of *Peranzana Alta Daunia* olives at the end of fermentation, suggesting that some indices (technological traits of lactobacilli, ratio yeasts vs. LAB, abundance of non-*Saccharomyces* yeasts) could be useful to define a microbiological profile of the variety.

## 1. Introduction

According to the International Olive Oil Council (IOOC) [1], table olives are defined as “the product prepared from the sound fruits of varieties of the cultivated olive trees (*Olea europaea* L.) that are chosen for their production of olives whose volume, shape, flesh-to-stone ratio, fine flesh, taste, firmness, and ease of detachment from the stone make them particularly suitable for processing”. Among fermented foods, table olives have a big global socio-economic impact: according to the report of the IOOC, during the last season, world production was close to 2,700,000 tons, with the main producers in the European Union (EU), particularly Mediterranean countries (Spain, Greece, Italy and Portugal) [2]. European consumption was also considerable, amounting to about 578,000 tons (Spain is the main consumer) [3].

*Peranzana Alta Daunia*, also called *Provenzana* or *Provenzale*, is an olive cultivar whose production area is the north-west of the province of Foggia in the Apulia region of Italy; the cultivar is mostly present in a limited area of Apulia called “Tavoliere delle Puglie”, and is identifiable, among others, in the cities of San Paolo di Civitate, San Severo and Torremaggiore. *Peranzana Alta Daunia* is not hybridized and is a pure cultivar; thanks to the excellent consistency of its pulp and its sweet and balanced taste, it is an excellent table olive, especially if prepared through the Greek style. Among the three main methods applied for olive ripeness (Spanish style, oxidation; Californian style, natural; or Greek style), the last is generally used for *Peranzana Alta Daunia* olives, which are left to ferment in brine without any preliminary treatment with NaOH, whose primary scope is to remove oleuropein, a phenolic compound that confers bitterness [4].

The biota of table olives is complex and variable, since it can be affected by several factors, such as pH, water activity, sugar availability, concentration of phenolic compounds, temperature, oxygen availability, salt, etc. [5,6]. However, lactic acid bacteria (LAB) and yeasts can be considered the dominant microbial groups: the first are the main players of fermentation through acidification of the brine and consequent inhibition of other undesirable microbial groups (which are not able to tolerate low pHs), and the latter give a precious contribution to sensorial quality improvement (in terms of taste, odor and flavor) due to their capacity to produce volatile organic compounds [7,8]. In addition, some studies [9,10] have underlined that yeasts are able to stimulate LAB growth through a two-way action: (1) production of crucial compounds for their growth, such as vitamins, amino acids, nitrogen bases, etc.; (2) degradation of some inhibitors, such as phenolic compounds [10,11]. In general, the ratio between LAB and yeasts is correlated on the type of processing method [12], and the prevalence of one or another microbial group is a determinant for producing an optimal product in terms of both organoleptic characteristics and shelf life [13].

In this context, the present study was focused on the microbiological profile of *Peranzana Alta Daunia* olives cultivated in different areas, since, at present, information on these aspects is still lacking; this is particularly relevant in potentially obtaining the protected denomination origin (PDO) already assigned to two other Italian table olive cultivars, such as Bella di Cerignola (Bella della Daunia olives variety Bella di Cerignola’, Regulation EC No 1904/2000) and Nocellara del Belice (Regulation EC No 134/1998).

## 2. Materials and Methods

### 2.1. Sampling

Samples of brines of *Peranzana Alta Daunia* table olives produced in eight different areas were collected and analyzed at the end of the fermentation process (6 months after brining; the initial concentration of NaCl was 10–12%) in two different years (2014 and 2017) (Table 1). All analyses were carried out in duplicate.

For microbiological analyses, brines were serially diluted and plated in triplicate for enumeration of (1) lactic acid bacteria (LAB) on MRS agar, supplemented with 0.17 g/L cycloheximide (Sigma-Aldrich, Milan, Italy), incubated at 30 °C under anaerobic conditions for 48–72 h; (2) yeasts on Yeast Peptone Glucose Agar (bacteriological peptone 20 g/L, yeast extract 10 g/L, glucose 20 g/L, agar 15 g/L), and on WL nutrient medium to discriminate between *Saccharomyces* and non-*Saccharomyces* yeasts; both media were supplemented with 0.1 g/L chloramphenicol (C. Erba, Milan, Italy) and incubated at 25 °C for 2–4 days. Yeast attribution to *Saccharomyces* and non-*Saccharomyces* groups was also confirmed through microscope examination and streaking on Lysine Agar Medium; (3) total aerobic count on PCA (Plate Count Agar), incubated at 30 °C for 24–48 h; (4) staphylococci on Mannitol Salt Agar and on Baird Parker Agar Base (for coagulase-positive cocci), both incubated at 37 °C for 24–48 h; (5) clostridia on SPS agar, incubated under strictly anaerobic conditions at 37 °C; (6) enterobacteria on Violet Red Bile Glucose Agar (VRBGA), incubated at 37 °C for 18–24 h. All media and supplements were purchased from Oxoid (Basingstoke, UK).

The pH of brine was measured using a Crison pH meter (Crison Instruments, Barcelona, Spain), whereas salt amount was evaluated by means of refractometer Sper Scientific model 106 ATC (Scottsdale, AZ, USA).

### 2.2. LAB Growth Assays

Presumptive LAB (confirmed through microscope examination, catalase test, Gram staining, acidification and lactic acid production) were isolated from plates, purified, and stored in MRS broth, supplemented with 33% sterile glycerol at −20 °C. Before each experiment, LAB were grown in MRS broth, incubated at 30 °C for 24 h.

These assays were conducted in MRS broth supplemented with different amounts of NaCl (6%, 8%, and 10%) or incubated at different temperatures (10 and 45 °C). All samples were inoculated to 3 log CFU/mL with the tested strains (134 isolates), incubated at given temperatures, and the growth was evaluated after 24 and 48 h at an absorbance of 600 nm using a UV–Vis DU 640 Beckman spectrophotometer (Fullerton, CA, USA). Samples of non-modified MRS broth, inoculated and incubated at 30 °C, were used as controls.

### 2.3. Modeling

Data from growth assays for lactobacilli were modeled as Growth Index (GI) [14], a relative index that compares the growth in an experimental condition as a function of the optimal growth; it is not a growth or a fitting curve, but a time-dependent index pinpointing if, in a certain amount of time, the microorganism is inhibited or not, compared to the optimal conditions (control). If its value is <25%, growth is completely inhibited, whereas if it is >75%, growth is not inhibited; intermediate values indicate partial inhibition.

Data of GI at 48 h (median value of each area) were used as input for a two-way joining analysis using the software Statistica for Windows, version 12.0 (Statsoft, Tulsa, OK, USA); the amalgamation method was based on the single linkage approach and percent disagreement.

Two-way joining is a clustering approach with some similarities to cluster and principal component analyses; it combines cases and samples (in this research sample origin) using a set of input variables; the output is clustering at the global level, but also semi-quantitative results for each variable.

## 3. Results and Discussion

In this study, brines of naturally fermented *Peranzana Alta Daunia* table olives produced in eight different areas of Southern Italy (see Table 1) were microbiologically analyzed at the end of a spontaneous fermentation process. In general, when olives are processed with the Greek (or natural) method, fruits are washed and directly immersed in a brine solution, without any treatment with NaOH [15,16]. The bitterness of the olives is lost because the oleuropein disperses into the brine and undergoes posterior acid hydrolyzation [15,16]. Due to the high salt concentration used, the fermentation is driven mainly by yeasts, and then by LAB; thus, these microbial groups constitute the dominant biota from the middle of the fermentation and thereafter [17,18,19,20,21,22,23,24].

After sampling, the NaCl of brines was between 7.9 and 10.1%, while pH was 4.3–4.6. Focusing on the microbiological profile, clostridia were always below the detection limit, enterobacteria were found only in two samples (2.1–3.2 log CFU/mL), staphylococci were between 3.1 and 4.23 log CFU/mL, and coagulase-positive cocci were never detected.

Total aerobic count was between 4 and 7.3 log CFU/mL, while LAB were from 1.6 log CFU/mL (sample Q) to 4.3 log CFU/mL (sample G) and yeasts from 4.3 to 6.4 log CFU/mL.

**Figure 1:**
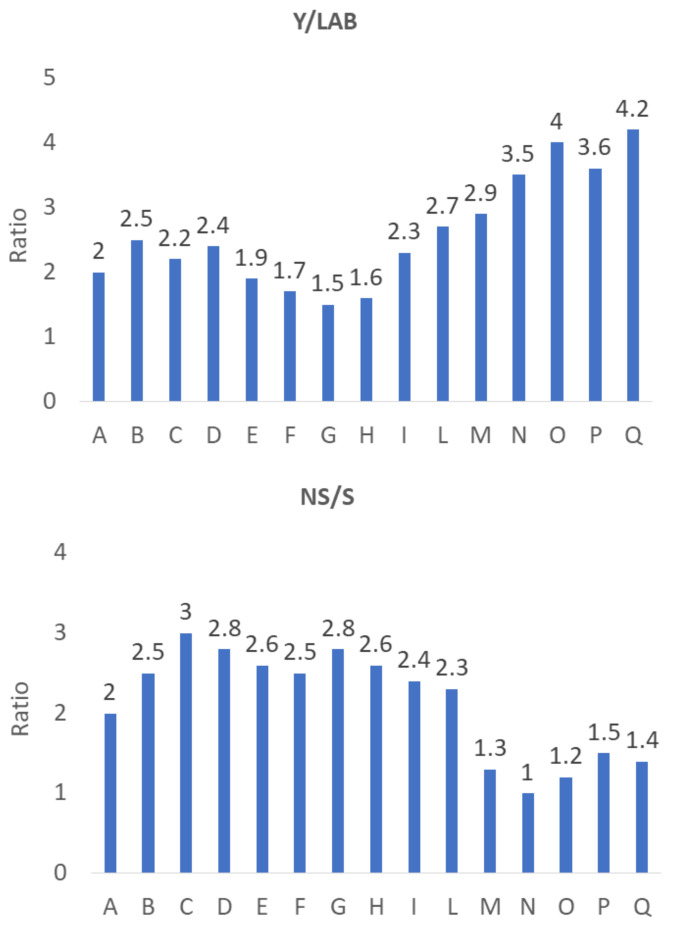
Ratio between yeasts and lactic acid bacteria (Y/LAB) and between “non-*Saccharomyces*” and “*Saccharomyces*” strains (NS/S) in table olives collected in eight different areas at the end of a natural and spontaneous fermentation process. For the meaning of codes A-Q see Table 1.

Apart from the raw counts, for a correct understanding of the microbiological profile of a matrix, it is important to focus on the relative abundances of some groups; for olives, one of the most important parameters is the ratio yeasts/LAB, which is dependent on the type of processing [12]; namely, LAB are dominant and drive fermentation in Spanish-style processing, while yeasts are predominant in other styles. This difference is due to the dispersion of phenolic compounds into the brine, which strongly inhibit the LAB population in processes not using NaOH to debitter [25,26], such as the Greek method. The results obtained in this investigation confirmed this trend and highlighted a prevalence of yeast strains on LAB, with some differences in the ratios of Y/LAB, as shown in Figure 1. Namely, a ratio ranging from 1.6 to 2.5 was found in the samples collected in the areas of Torremaggiore, San Severo, Apricena and Lucera (codes from A to H), 2.3 and 2.7 in San Paolo di Civitate (codes I and L), and 2.9 in Gargano (code M), highlighting a better equilibrium among yeasts and LAB than that observed in samples from Termoli and Serracapriola (Y/LAB 3.5–4.2, codes from N to Q).

Figure 1 also shows the ratio between “non-*Saccharomyces*” and “*Saccharomyces*” strains (NS/S); in this case, a ratio of 2.8–3 was observed for the samples collected in the areas of San Severo (C, D) and Lucera (G), thus highlighting a prevalence of non-*Saccharomyces* yeasts for these batches, while the ratio was lower in the samples A (2), M, O, Q, N and P (1–1.5) (similar counts of *Saccharomyces* vs. non-*Saccharomyces*). The prevalence of non-*Saccharomyces* yeasts was also found by other authors who isolated strains mainly belonging to the *Candida* genus from table olives and their brines [27,28,29,30,31,32,33,34,35], while in other olives (*Negrinha de Freixo* in Portugal), with a processing protocol similar to *Peranzana Alta Daunia* table olives, a correct fermentation was related to the recovery of both *Saccharomyces* and non-*Saccharomyces* yeasts in the last days of fermentation (after 5 months) [36].

In the research of Mujdeci et al. [37], the effects of regional differences on yeast biota of Turkish olives (*Gemlik* cultivar) processed through the natural style were investigated; the results showed the existence of 12 different species, mainly non-*Saccharomyces*, with *Candida famata* and *C. pelliculosa* being the most representative species. A higher presence of non-*Saccharomyces* species was also found on the biota of green *Manzanilla* cv. olives [38]; there is also evidence of the presence of the genera *Bullera*, *Dekkera*, *Sporobolomyces*, *Lachancea*, *Citeromyces* and *Rhodotorula* [28,32].

The role of yeasts in table olive fermentation is a recent research topic, because in the past they were always regarded as spoilers and only in 2008 was their positive role highlighted, above all for some enzymatic traits and for the influence on the sensory scores [39]. As reported above, both *Saccharomyces* and non-*Saccharomyces* yeasts are found in table olives, and their recovery could have different meanings. For example, some non-*Saccharomyces* yeasts could contribute to the biodegradation of phenols, and to the production of volatile compounds, while *S. cerevisiae* strains could promote the growth of LAB, thus indirectly favoring brine acidification. However, it is worth mentioning that both *Saccharomyces* and non-*Saccharomyces* yeasts could have negative roles (for example, fruit softening due to polygalactunorase activity, lactic acid respiration for non-*Saccharomyces* yeasts or CO_2_ production in the headspace of packed products for *Saccharomyces* spp.) [39]. The definition of the exact role of yeasts depends on the olive variety, as well as on the production stage (a yeast species could be positive during fermentation and negative in packed samples). Although preliminary and with few data available, the ratio of non-*Saccharomyces*/*Saccharomyces* could be labeled as the first measure to define the biodiversity of *Peranzana Alta Daunia* table olives, and higher recovery of non-*Saccharomyces* could be related to the peculiar sensory traits of this kind of olives.

Focusing on LAB, by a preliminary examination, 1136 lactobacilli were recovered from plates, as shown in Table 1. All these isolates were Gram positive, catalase negative, rod-shaped, and able to reduce pH in a lab medium.

Many lactobacilli can grow on olives, but *Lactiplantibacillus plantarum* is considered the most relevant actor of fermentation, so much so that it is generally suggested for use as starter culture in single or mixed combinations [7]. Besides *Lactiplantibacillus*, other genera often recovered from table olives are *Pediococcus*, *Leuconostoc* and *Enterococcus* [13,23,40,41].

In the second step of this research, the attention was focused only on 134 LAB isolates, selected from all isolates recovered from samples (see Table 1), based on their technological robustness (survival during freezing, MRS broth acidification after 48 h to pH 4.5, growth at least with 6% NaCl). Isolates were studied in relation to their ability to grow at 10 and 45 °C, and with salt added (6, 8 and 10% NaCl). The data were modeled with the equation of Growth Index (GI): a GI *>* 75% suggests that the stress condition does not affect the kinetic growth of the target strains, whereas a GI *<* 25% or in the range 25–75% highlights strong or partial inhibition, respectively.

**Figure 2:**
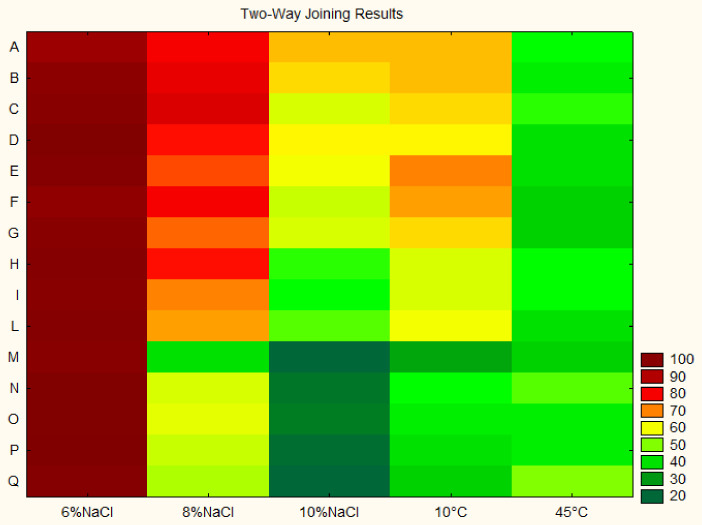
Growth Index at 48 h (%) (median value) of studied strains (134 presumptive *lactobacilli*) in MRS broth incubated under stressing conditions (presence of NaCl or temperature of 10 and 45 °C) after 24 h. The input values for two-way joining are in Supplementary Table S1. For the meaning of codes A-Q see Table 1.

The results (Figure 2) suggest that all the isolates from the samples collected in the areas of Torremaggiore, San Severo, Apricena, Lucera and San Paolo di Civitate (samples from A to L) were generally more resistant to stress conditions; in fact, for these strains, GI values ranging from 68 to 82% and from 47 to 65% were recorded during the growth in the presence of 8% and 10% of salt, respectively, thus confirming the high technological resistance in relation to NaCl of some indigenous isolates of the natural biota of directly brined olives [42]. On the other hand, lower GI values were measured for the isolates from the areas of Gargano, Termoli and Serracapriola, for which GI values of about 20–25% were estimated in the presence of the higher salt concentration (10% NaCl). Regardless of the origin, all the isolates showed a delay during the growth at 45 °C, while more vigorous growth (GI about 60%) was recorded at 10 °C only for the isolates from the areas A–L. The assays to test the ability to grow at different temperatures are crucial in a characterization study. In fact, especially in Southern Italy, the fermentation of table olives takes place from the end of September to the first days of December; consequently, the ability to grow both at low (10 °C) and high (37 °C) temperatures suggested that they could be able to colonize the olive surface during the fermentation process.

Although traditionally related to Spanish-style olives, LAB could also play an important role in untreated olives, in terms of acidification, safety and aroma development with yeasts [42,43]. Thus, these results, along with some other data available in the literature [44,45], could be of interest when designing and selecting a combined starter of yeasts/lactic acid bacteria from the natural biota of *Peranzana Alta Daunia* table olives.

## 4. Conclusions

This paper represents the first preliminary approach to define the microbiological profile of naturally fermented *Peranzana Alta Daunia* table olives. Although further investigations are required, some key points can be pointed out:

- It is possible to individuate two macro-areas: one including the areas of Torremaggiore, San Severo, Apricena, Lucera and San Paolo di Civitate (area 1), and another comprising Gargano, Termoli and Serracapriola (area 2);
- A prevalence of yeast strains on lactic acid bacteria was common in both zones, but a better equilibrium among these microbial groups was generally found in the samples collected in area 1 (lower Y/LAB ratio);
- A higher abundance of “non-*Saccharomyces*” yeasts was found in the samples from San Severo and Lucera;
- LAB isolates from area 1 were more resistant to stress conditions, being able to grow both at 10 °C and in the presence of 10% of salt.

In conclusion, this paper offers a first insight into the microbiological profile of *Peranzana Alta Daunia* olives at the end of fermentation, suggesting that some indices (technological traits of lactobacilli, ratio yeasts vs. LAB, abundance of non-*Saccharomyces* yeasts) could be used in the future to identify possible sub-areas in the production zones, probably linked to production practices or pedo-climatic characteristics.

## Figures and Tables

**Table 1:** Origin of brines’ samples analyzed throughout this research: code, year of sampling, samples’ origin, and number of lactobacilli recovered and studied for each location.

Samples Analyzed in 2014	Samples Analyzed in 2017	Samples’ Origin	Isolates of Lactobacilli Recovered	Isolates of Lactobacilli Studied
A	B	Torremaggiore, Foggia, Apulia	174	19
C	D	San Severo, Foggia, Apulia	192	19
E	F	Apricena, Foggia, Apulia	178	19
G	H	Lucera, Foggia, Apulia	172	20
I	L	San Paolo di Civitate, Foggia, Apulia	150	15
M		Gargano, Foggia, Apulia	56	8
N	O	Termoli, Campobasso, Molise	108	16
P	Q	Serracapriola, Foggia, Apulia	106	18
		Tot.	1136	134

## Data Availability

The data presented in this study are available on request from the corresponding author.

## Supplementary Materials

The following supporting information can be downloaded at: https://www.mdpi.com/article/10.3390/foods11142100/s1, Table S1: Median value of Growth Index values at 48 h of lactobacilli in presence of NaCl (6, 8, and 10%), at 10 and 45 °C. Input data for two-way joining reported in Figure 2.
