# Supplementary material for: A Preliminary Approach to Define the Microbiological Profile of Naturally Fermented Peranzana Alta Daunia Table Olives
Source: Foods. 2022 Jul 14;11(14):2100. doi: 10.3390/foods11142100 (PMC9315826; doi:10.3390/foods11142100)
Supplement: Supplementary file 1 [file foods-11-02100-s001.zip › foods-1798310-supplementary.pdf]

Table S1. Median value of Growth Index values at 48 h of lactobacilli in presence of NaCl (6, 8, and 10%), at 10 and 45°C. Input data for two way-joining reported in Figure 2.

	GI-6%NaCl	GI-8% NaCl	GI-10%NaCl	GI-10 °C	GI-45 °C	Number of isolates used for the experiments
A	93	80	65	65	44	10
B	97	82	63	66	42	9
C	99	83	56	64	45	8
D	103	78	62	61	39	11
E	102	74	59	69	39	7
F	95	79	54	68	38	12
G	99	71	55	64	37	9
H	101	77	45	56	43	11
I	100	69	43	55	44	7
L	102	68	47	60	39	8
M	100	39	18	32	38	8
N	103	55	23	43	47	9
O	104	58	25	42	42	7
P	105	54	22	39	41	6
Q	102	52	18	37	49	12
